# A Cryptic Non-Inducible Prophage Confers Phage-Immunity on the *Streptococcus thermophilus* M17PTZA496

**DOI:** 10.3390/v11010007

**Published:** 2018-12-22

**Authors:** Vinícius da Silva Duarte, Sabrina Giaretta, Stefano Campanaro, Laura Treu, Andrea Armani, Armin Tarrah, Sérgio Oliveira de Paula, Alessio Giacomini, Viviana Corich

**Affiliations:** 1 Department of Microbiology, Universidade Federal de Viçosa, Av. Peter Henry Rolfs, s/n, Campus Universitário, Viçosa-MG 36570-900, Brazil; vinicius.dasilvaduarte@unipd.it; 2 Department of Agronomy Food Natural Resources Animals and Environment, University of Padova, 35020 Legnaro, Italy; sabry.giaretta@gmail.com (S.G.); tarrah.armin@gmail.com (A.T.); alessio.giacomini@unipd.it (A.G.); viviana.corich@unipd.it (V.C.); 3 Department of Biology, University of Padova, 35121 Padova, Italy; stefano.campanaro@unipd.it; 4 Venetian Institute of Molecular Medicine, 35129 Padova, Italy; andrea.armani@unipd.it; 5 Department of General Biology, Universidade Federal de Viçosa, Viçosa-MG 36570-900, Brazil; sergio.oliveira.paula@gmail.com

**Keywords:** *Streptococcus thermophilus*, lipoprotein (Ltp), noncoding region, bacteriophages, cryptic prophage

## Abstract

*Streptococcus thermophilus* is considered one of the most important species for the dairy industry. Due to their diffusion in dairy environments, bacteriophages can represent a threat to this widely used bacterial species. Despite the presence of a CRISPR-Cas system in the *S. thermophilus* genome, some lysogenic strains harbor cryptic prophages that can increase the phage-host resistance defense. This characteristic was identified in the dairy strain *S. thermophilus* M17PTZA496, which contains two integrated prophages 51.8 and 28.3 Kb long, respectively. In the present study, defense mechanisms, such as a lipoprotein-encoding gene and Siphovirus Gp157, the last associated to the presence of a noncoding viral DNA element, were identified in the prophage M17PTZA496 genome. The ability to overexpress genes involved in these defense mechanisms under specific stressful conditions, such as phage attack, has been demonstrated. Despite the addition of increasing amounts of Mitomycin C, M17PTZA496 was found to be non-inducible. However, the transcriptional activity of the phage terminase large subunit was detected in the presence of the antagonist phage vB_SthS-VA460 and of Mitomycin C. The discovery of an additional immune mechanism, associated with bacteriophage-insensitive strains, is of utmost importance, for technological applications and industrial processes. To our knowledge, this is the first study reporting the capability of a prophage integrated into the *S. thermophilus* genome expressing different phage defense mechanisms. Bacteriophages are widespread entities that constantly threaten starter cultures in the dairy industry. In cheese and yogurt manufacturing, the lysis of *Streptococcus thermophilus* cultures by viral attacks can lead to huge economic losses. Nowadays *S. thermophilus* is considered a well-stablished model organism for the study of natural adaptive immunity (CRISPR-Cas) against phage and plasmids, however, the identification of novel bacteriophage-resistance mechanisms, in this species, is strongly desirable. Here, we demonstrated that the presence of a non-inducible prophage confers phage-immunity to an *S. thermophilus* strain, by the presence of *ltp* and a viral noncoding region. *S. thermophilus* M17PTZA496 arises as an unconventional model to study phage resistance and potentially represents an alternative starter strain for dairy productions.

## 1. Introduction

The thermophilic Lactic Acid Bacterium (LAB) *Streptococcus thermophilus* is an extremely important starter culture, in the dairy industry, for production of cheeses and yogurts [1,2]. Its technological use is mostly linked to its ability to quickly acidify the substrate, a very important feature, since it is known that a pH decrease leads to modifications in bacterial [3,4] and also yeast [5,6] population composition. Its use in the dairy industry has an average market value of US$ 40 billion [7]. *S. thermophilus* starter cultures in the dairy environment are constantly threatened by bacterial viruses (bacteriophages or phages), which are the most abundant biological entities in the biosphere [8,9]. The lysis of starter culture cells leads to relevant economic losses, by lowering the quality of the end product, or even leading to a total process failure [10,11]. To overcome this problem, the dairy industry has adopted many different strategies to limit phage attacks against bacterial starter cultures [12]. During the past decades, molecular and genomic approaches have revealed diverse genetically defined resistance mechanisms directed against *Streptococcus* phages [13,14,15,16]. It was reported that *S. thermophilus* strains have natural adaptive immunity against phages, including CRISPR-Cas, Ltp lipoprotein, and noncoding viral DNA elements [15,16,17,18]. The last two mechanisms are part of the temperate bacteriophage driven immunity that protects the host (as well, as the prophage) from lysis, by blocking DNA injection and replication, respectively [15,17]. The phage-encoded lipoprotein Ltp is the prototype of a widely-distributed family of cell-surface-exposed lipoproteins, involved in superinfection exclusion (sie). The *ltp* gene belongs to the “moron” class, which is characterized by a common strategy to confer host benefits through phage-mediated horizontal gene transfer (HGT), by blocking invader DNA injection [15,19,20]. The *ltp* gene is usually located within the lysogeny module and is constitutively transcribed, while the virus is in the prophage state [15]. A different immunity system, encoded by prophages, is based on a mechanism determined by the increased copy number of a viral noncoding region, with characteristics of an origin of replication (*ori*) sequence. This region, that can block the accumulation of the invading phage DNA, was found for the first time in the DNA replication module of phage ΦSfi21 [17]. As previously demonstrated by Lamothe et al. (2005) [21], the transcription of *cro-ori* regions start 5 min after phage infection.

Due to the presence of these defense mechanisms in *S. thermophilus*, the identification of bacteriophage-insensitive starter cultures is extremely relevant and has significant economic importance. Additionally, the increasing amount of genomic data available on *S. thermophilus* phages can facilitate the identification of bacteriophage superinfection exclusion and the development of new resistance mechanisms. The discovery of new immune mechanisms against viral infection in *S. thermophilus* will help in the development of innovative strategies, to protect bacteria from phage attack. Here, we present the genomic analysis of two cryptic prophages, which naturally infected *S. thermophilus* M17PTZA496, a well-characterized strain [22,23,24] possessing interesting in vitro probiotic properties, along with anticancer activity and folic acid production [25]. The combined presence of two prophages features associated with phage immunity, i.e., *ltp* and a viral noncoding region in the same prophage, was investigated. Moreover, we evaluated the activity of these two immune mechanisms under mitomycin C (MmC) stress and phage attack conditions.

## 2. Materials and Methods

### 2.1. Strain and Growth Conditions

The bacterial strain *S. thermophilus* M17PTZA496 was isolated in the Valle d’Aosta Region (Italy) from Fontina, a protected designation of origin (PDO) cheese. This isolate was stored, as a frozen stock (−80 °C), in sterile reconstituted (10%, *w*/*v*) commercial nonfat skim milk, supplemented with 15% (*v*/*v*) glycerol. *S. thermophilus* M17PTZA496 was routinely grown at 44 °C, for 24 h, in modified M17 medium containing lactose 0.5% (*w*/*v*) (th-LM17) [26].

### 2.2. Bioinformatics Analysis

Following genomic analysis and strain comparison [27,28], a more detailed investigation was conducted by analyzing the prophages in the *S. thermophilus* M17PTZA496 chromosome. For this purpose, PHAge Search Tool Enhanced Release (PHASTER) [29] was used to identify and annotate all viral sequences. Functional information was obtained using UniProt and Pfam databases [30] and by manual refinement. Viral genome alignment visualization and manual inspection were performed using Progressive MAUVE [31]. The CGView Server [32] was used to generate a graphical map of the TP1-M17PTZA496 genome.

For comparative analysis, eighty-three *S. thermophilus* bacteriophages and five *Lactococcus lactis* phages whole genome sequences, downloaded from the National Center for Biotechnology Information (NCBI) database (Table S1), were used. A fragmented all-against-all comparison in the TBLASTX mode was performed with the Gegenees 2.0.0 software [33], setting the parameters to 50/25 (fragment-size/slide-size), as described by Barylski et al. (2014) [34]. A heat plot was generated by setting the maximum threshold (40%), in order to obtain the best phylogenomic overview. Unrooted phylogenetic tree was computed, using the SplitsTree4 following the neighbor joining method [35]. A whole genome comparison with *pac*-type phages from the genus Sfi11virus, TP1-M17PTZA496, and Streptococcus phage 20617, was performed, using the Easyfig comparison tool [36,37,38]. Finally, CRISPRdb [39] and HostPhinder [40] tools were used to predict the potential bacterial hosts of the identified prophages. The nucleotide sequence of *S. thermophilus* M17PTZA496 was taken from the GenBank database (accession No. NZ_CM002372.1).

### 2.3. S. thermophilus M17PTZA496 Prophage Induction Assay, DNA Extraction, Semi-Quantitative PCR, and Transmission Electron Microscopy

The prophage induction assay was conducted at a small scale (96-well microplates), as described by Oliveira et al. (2017) [41] and Arioli et al. 2018 [36]. Briefly, *S. thermophilus* M17PTZA496 was grown at 44 °C for 24 h, in th-LM17. The culture was then diluted with sterile th-LM17 medium to reach an optical density at 600 nm (OD_600_) of, approximately 0.1. A final volume of 200 µL was inserted into the wells of a 96-well microplate. In the chemically-treated groups, four phage-inducing agents (MmC −1, 2, 3, and 4 µg/mL; nalidixic acid −0.1, 0.2, and 0.4 µg/mL; NaCl −100, 200, and 400 mM, and H_2_O_2_ −100, 200, and 400 mM) were added to the early log-phase cultures. To evaluate the effect of low (0.1, 0.25, and 0.5% *w*/*v*) and high concentration (1.0% *w*/*v*) of lactose and sucrose on *S. thermophilus* M17PTZA496 growth curve and prophage induction, cells were prepared, as described previously, washed three times with the phosphate buffered saline (PBS: KH_2_PO_4_ 144 mg/L, NaCl 9 mg/L, Na_2_HPO_4_·7H_2_O 795 mg/L, pH 7.4) and resuspended in the M17 medium, modified with lactose and fructose, at different concentrations, to reach an OD_600_ of 0.1. Then aliquots of 200 µL were transferred into the wells of a 96-well microplate. Measurements were automatically obtained by a Spark 10M (Tecan Trading AG, Männedorf, Switzerland) every 30 min. The entire assay was carried out in triplicates.

A semiquantitative PCR test was used to estimate the amount of viral DNA in the bacterial cells and the integrity of the virus attachment sites. Sampling was performed after 0, 30, 60, and 90 min of bacterial growth and for each MmC concentration used. Bacterial DNA extraction was performed by adding 500 µL of bacterial culture to 50 µL of lysis buffer (NaOH 0.05 M; SDS 0.25%) in a 1.5 mL tube, mixed by vortexing for 2 min and incubated at 94 °C, for 15 min to achieve cell lysis. Cell debris was removed by centrifugation at 4,500× *g* for 10 min. DNA yield and purity were assessed by NanoDrop (NanoDrop 2000c, Thermo Scientific, Waltham, MA, USA).

Four primer couples were manually designed inside the coding region of the major capsid protein, in the attachment sites *att*L/*att*R and in a M17PTZA496 internal control region (Table 1), using the online tool NCBI/Primer-BLAST [42]. Primers were obtained from Invitrogen (Thermo Fisher Scientific, Rodano, MI, Italy). PCR reactions were performed using PureTaqTM Ready-To-Go^TM^ PCR Bead kit (GE Healthcare, Munich, Germany), using 50 ng of bacterial DNA as the template.

The presence of complete or defective viral particles in treated and untreated-MmC *S. thermophilus* cultures was evaluated by TEM, at different time points, namely 0, 30, 60, and 90 min. Bacterial cultures (1 mL) were harvested by centrifugation at 5,500× *g* for 15 min, at 4 °C, and the pellet was resuspended in 100 µL of SM buffer (5.8 g NaCl, 2.0 g MgSO_4_·7H_2_O, 50 mL Tris-HCl 1M, 5 mL gelatin 2%, pH 7.5 and H_2_O to 1 L). Chloroform (10% *v*/*v*) and NaCl 1M (final concentration) were added, in order to provoke cell lysis and release of viral particles. After 2 h of incubation at 4 °C, cellular debris were removed by centrifugation at 14,000× *g* for 20 min, at 4 °C, the supernatant was collected and transferred to the observation Formvar-coated grids and negatively stained with 2% (*w*/*v*) uranyl acetate. A transmission electron microscope (Tecnai 12, FEI Thermo Fisher Scientific, Eindhoven, The Netherlands), operating at an acceleration voltage of 80 kV, was used to examine the samples. Images were acquired at a resolution of 50 nm.

### 2.4. Phage-Susceptibility Screening, and Phage-Binding Assay

Six bacteriophages, namely, vB_SthS-VA214 [44], vB_SthS-VA353, vB_SthS-VA444, vB_SthS-VA460 [44], vB_SthS-VA698, and vB_SthS-VA720, were previously characterized, considering their variable region 2 (VR2) [45], and were selected and tested for their ability to infect *S. thermophilus* M17PTZA496. Bacteriophages were kindly provided by the Istituto per la Qualità e le Tecnologie Agroalimentari, Veneto Agricoltura (Thiene, Italy). Using a multiplicity of infection of 0.1, the six bacteriophages were independently added to a *S. thermophilus* M17PTZA496 growing culture (OD_600_ 0.3), in th-LM17, supplemented with 1% CaCl_2_ 1M. After incubation at 44 °C for 30 min, MmC (1 µg/mL) was added and samples were incubated for a further 30 min. Cells were then collected by centrifugation at 12,000× *g* for 4 min, at 4 °C, and the supernatant used for viral titration. Briefly, an aliquot of 100 µL was serially diluted (1:10) in SM buffer and each dilution was mixed with 0.2 mL of a host culture, previously grown overnight [46]. To this mix, 35 µL of CaCl_2_ 1 M and 4 mL of molten th-LM17 soft agar (agar 0.75%) were added and poured onto a th-LM17 agar base plate (agar 1.5%), containing 1% CaCl_2_ [47]. Plates were incubated at 37 °C for 24 h. Viral titer was expressed in terms of plaques forming units per milliliter (PFU/mL). The percentage of phage particles bounded to *S. thermophilus* M17PTZA496 and the rate of attachment were calculated, as previously reported [48,49].

### 2.5. Transcriptional Activity Assay

An *S. thermophilus* M17PTZA496 overnight culture (OD_600_ 1.4) was adjusted to an OD_600_ of 0.1 in 300 mL of th-LM17, containing 10 mM CaCl_2_ and 10 mM glycine, and incubated at 45 °C [45]. When the culture reached an OD_600_ of 0.3 (that was considered T0), 9 mL were transferred to two separate 15 mL polypropylene tubes, in which 1 mL of PBS (defined as Cntr1) and 1 mL of a vB_SthS-VA460 suspension (~10^7^ PFU/mL) (defined as Pt1) were added, respectively. Tubes were incubated at 45 °C for 30 min. After this period, defined as T1, MmC (1 µg/mL) was added to both tubes (defined Cntr2 and Pt2, respectively) and a final incubation at 45 °C for 30 min, identified as T2, was performed. At each sampling time (T0, T1, and T2), a 100 µL aliquot was collected to determine the number of viable cells, using the drop plate methodology [50]. Viral titration was performed from Pt1 and Pt2 samples, as described above. All experiments were performed in triplicates. The experimental design is reported in Figure S1.

Samples obtained at T0, T1, and T2 were centrifuged at 4,500 g for 5 min, at 4 °C. The harvested cells were immediately frozen in liquid nitrogen and stored at −80 °C, to preserve RNA integrity. To perform cell lysis, 100 µL of lysozyme solution (10 mM Tris-HCl, 0.1 mM EDTA, 15 mg/mL lysozyme, pH 8.0) were added to the cell pellet and resuspended by vortexing. Afterwards, 0.5 μL of 10% (*w*/*v*) SDS were added, vortexed for 2 min and incubated for 5 min, at room temperature. After this, 350 μL of freshly prepared 1% (*v*/*v*) 2-mercaptoethanol lysis buffer and 50 mg of cold glass beads (diameter 0.6 mm, Sigma, St. Louis, MO, USA) were added and the samples were vortexed for approximately 2 min. Subsequently, 750 µL of TRIzol (Invitrogen, Rodano, Italy) were added, gently homogenized and incubated for 5 min, at room temperature. During this step, 1 µg of exogenous total RNA extracted from the skeletal muscle of *Mus musculus* was added to the sample, as an external housekeeping gene source, for real-time data normalization. Afterwards, 160 µL of chloroform were added, mixed, and incubated for 2 min, at room temperature. Following centrifugation at 12,000× *g* for 15 min at 4 °C, the colorless upper phase containing the RNA, was transferred to a fresh RNase-free tube containing 350 µL of isopropanol and incubated at −20 °C for 1 h. Tubes were then centrifuged at 12,000× *g* for 10 min at 4 °C, the pellet washed with 1 mL of 75% (*v*/*v*) ethanol and tubes centrifuged at 7,500× *g* for 5 min, at 4 °C, and left to dry at room temperature, for 5 min. After DNase I (Invitrogen, Rodano, Italy) treatment, the total RNA was purified using RNA Clean & Concentrator ™-5 (Zymo Research, Irvine, CA, USA), according to the manufacturer instructions.

RNA quality and quantity were verified by NanoDrop (ThermoFisher Scientific, Waltham, MA, USA), while RNA integrity was evaluated on denaturing formaldehyde agarose gel (0.5% *w*/*v*). The RNA quality obtained was good, according to the standard parameters for real-time expression analysis (Figure S2).

Primer pairs for the *cas7* (*csn2*), *ltp*, viral non-coding region, and terminase large subunit (Table 1) were designed using the on-line tool NCBI/Primer-BLAST [42] and manually checked on the PCR templates. Primers were obtained from Invitrogen (Thermo Fisher Scientific, Rodano, MI, Italy). The glyceraldehyde-3-phosphate dehydrogenase (*gapdh*) gene from *Mus musculus* was used as reference control [43].

One µg of RNA was reverse transcribed, using the RevertAid Reverse Transcriptase (Thermo Scientific) and random hexamer primers, according to the manufacturer recommendations. qRT-PCR was performed using Power SYBR^®^ Green PCR master mix (Life Technologies).

### 2.6. Statistical Analysis

Statistical analysis was performed with the GraphPad Instat 3 software (GraphPad, La Jolla, CA, USA), using the one-way analysis of variance (ANOVA), to evaluate the phage-binding capability and *S. thermophilus* M17PTZA496 viable cell counts, under different conditions. Assays were set up in triplicates and Tukey’s test was used as the *post hoc* test.

The relative expression level of each gene was normalized, taking into consideration the gene *gapdh* of *Mus musculus* as reference. Transcript levels from the RNA samples were evaluated using three technical replicates and the difference in the transcription of each specific gene was calculated with the 2^−ΔΔCT^ method [51]. Paired Student *t*-test was used to calculate the significance of the difference between the relative expression of the *cas7*, *ltp*, Siphovirus Gp157, and terminase large subunit, under different conditions. Statistically significant values were defined for *p* < 0.05.

## 3. Results

### 3.1. Genomic Analysis of the S. thermophilus M17PTZA496 Prophages

In the present study, a global genomic investigation was performed to describe the prophages identified in the *S. thermophiles*, from a taxonomic and functional perspective. The presence of two prophages in the *S. thermophilus* M17PTZA496 chromosome was predicted (Figure S3). According to their position in the bacterial genome they were named TP1-M17PTZA496 (bacterial genome position: 617,680–669,542 bp; 51.8 Kb in length) and TP2-M17PTZA496 (bacterial genome position: 890,763–919,156 bp). The estimated length of TP2-M17PTZA496 is 28.3 Kb, with a GC content of 34.74% and twenty-eight predicted protein-encoding genes. The TP2-M17PTZA496 gene annotation revealed some features with a relevant technological role, such as a methionyl-tRNA synthetase, a carotenoid biosynthetic protein, an exopolysaccharide associated protein (Eps4Q), a bacteriocin and a glucose transporter (Table S2). As described elsewhere, a temperate prophage can be considered non-inducible when the lysis module is not identified [52,53]. The same applies to TP2-M17PTZA496, even though it was classified as “intact” by the PHASTER software. For this reason, only TP1-M17PTZA496 was considered for further analysis.

**Figure 1:**
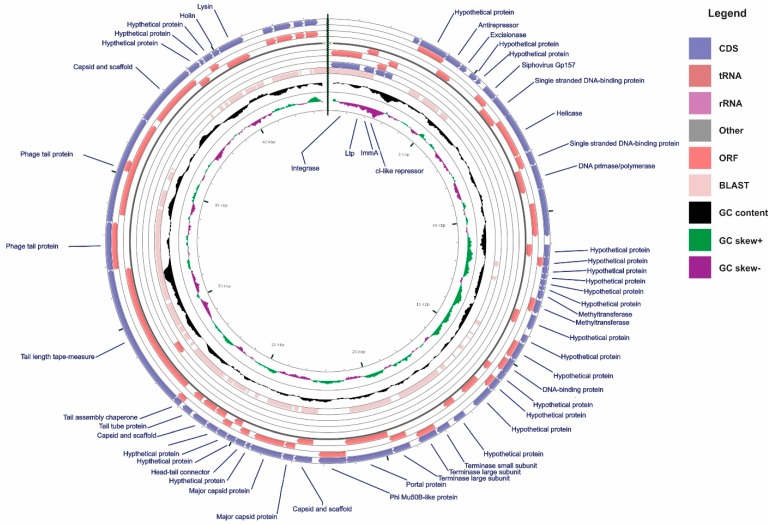
Genome map of the TP1-M17PTZA496. The linear genome was circularized to improve its visualization. CDS, ORF, BLAST against the *Streptococcus* phage 20617, GC content, GC skew+, and GC skew−, are reported in circles from the outside inwards. *Streptococcus* phage 20617 whole genome sequence was used as a reference for the BLAST analysis. Only the ORFs between the *attL* and *attR* are displayed for the TP1-M17PTZA496.

The genomic region representative of the TP1-M17PTZA496 has an estimated length of 51.8 Kb, a GC content of 39.13% and a total of sixty-one protein-encoding genes predicted between the *attL* and *attR* attachment sites (Figure 1). Results obtained from the similarity searches were manually curated, improving the overall annotation quality, and leading to the clarification of the functional role of integrated genes. Downstream to the left host-phage junction (*att*L), bacterial genes encoding enolase, glycogen synthase, and glycerate kinase were found, while upstream to the right junction (*att*R), bacterial genes encoding isoleucyl-tRNA synthetase, lipoteichoic acid synthase, methyltransferase, and 3-dehydroquinate dehydratase were identified. These integration sites have high similarity with those identified in the temperate bacteriophage Φ20617 [37], as revealed by the nucleotide BLAST analysis that showed 84% of identity between both viruses and a query cover of 76%. Presence of the tRNA synthetase genes in the *att* sites is a common feature, since tRNA coding regions have been recognized as integration site “hotspots” for viruses [54]. In the present study, isoleucyl-tRNA and methyonil-tRNA synthetase close to the *att* sites, showed this “preference rule” in the *S. thermophilus*.

In TP1-M17PTZA496, fifty-seven ORFs are transcribed from the positive strand. Most of the genes were assigned to putative functions and only seventeen ORFs remained as sequences coding for hypothetical proteins (Table S3). Some interesting features related to gene content and phage structure were identified; for example, ORF 2 (Ltp) and ORF 11, annotated as a gene with high similarity to the Gp157 of the *S. thermophilus* bacteriophages members of the genus *Sfi11virus*, commonly located in a *cro-ori* region, was found to be related to phage immunity. Regarding the lysogeny prophage module, ORF4 was annotated as CI-like repressor, while a *cro*-like coding sequence was missing.

**Figure 2:**
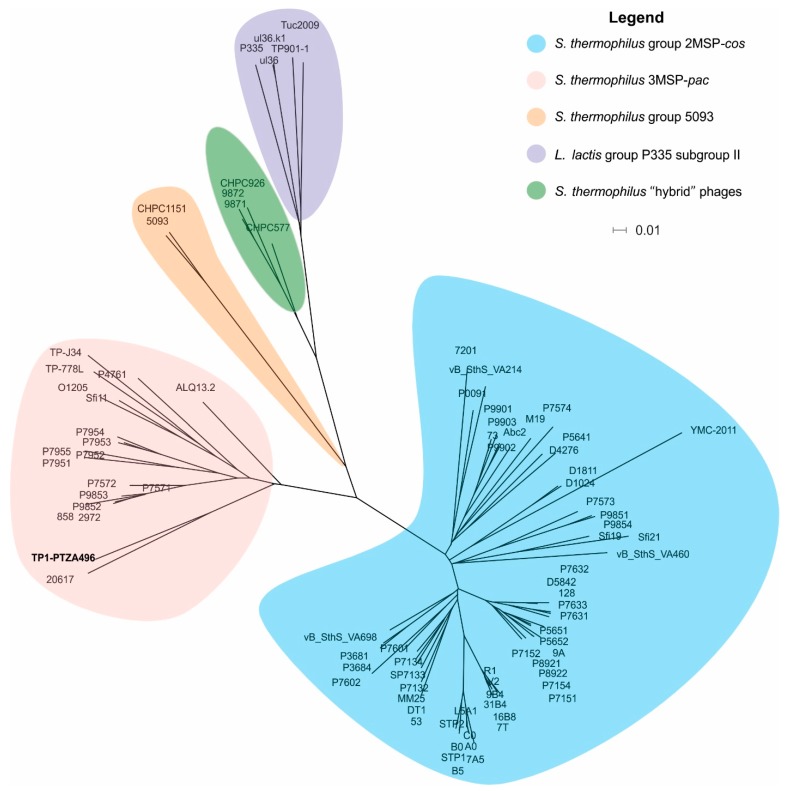
Phylogenomic tree constructed using the whole genome sequence of the TP1-M17PTZA496, 83 *S. thermophilus* and 5 *L. lactis* bacteriophages. GenBank accession numbers are reported in Table S1. The scale bar represents a 1% difference on the average tBLASTx score.

A phylogenomic tree was constructed, based on a “fragmented all-against-all comparison” with the aim of determining the taxonomic relationship that exist between prophage TP1-M17PTZA496 and other phages infecting the *S. thermophilus* and *Lactococcus lactis*. The phylogenetic tree evidenced that these viruses were clustered into five groups (Figure 2). According to this classification, the TP1-M17PTZA496 was associated with members of the *S. thermophilus* group 3MSP-*pac* and it is closely related to the temperate bacteriophage Φ20617 [14,37,54,55,56,57,58].

**Figure 3:**
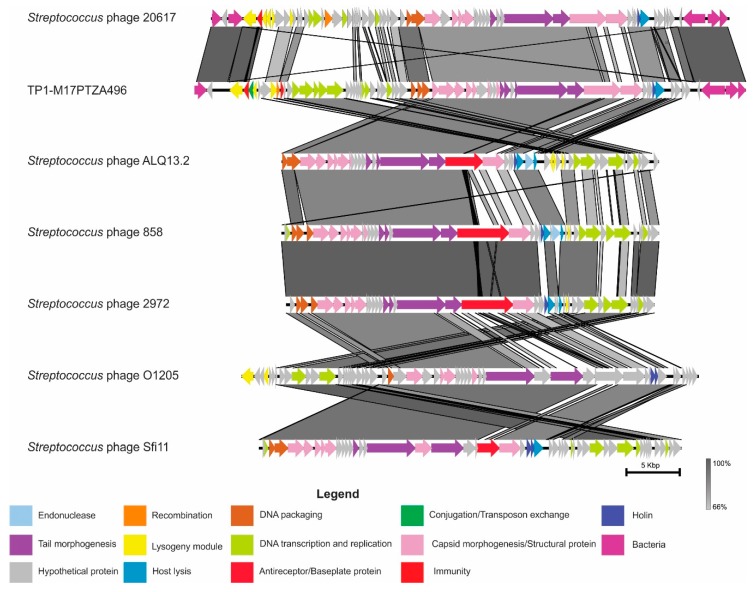
Sequence alignment among the *Streptococcus* phage 20617, TP1-M17PTZA496, and the members of the genus *Sfi11virus*. Gray shading corresponds to the percentage of identity of the nucleotide sequences.

According to the TP1-M17PTZA496 phylogenomic tree, the genome homology among the viruses grouped as 3MSP-*pac* was investigated, based on their translated nucleotide sequence (Figure 3). Despite this, the analysis revealed that the virus TP1-M17PTZA496 has a modular organization related to the virus φ20617, major differences were identified on the lysogeny module. The main changes are related to the absence of the coding sequence for *cro* and the presence of *immA*, which encodes an anti-immunity system involved in triggering, between the lytic and the lysogenic cycle [59]. The modules coding for structural proteins, tail morphogenesis, and host lysis (including holin) displayed a high similarity with other members of the *Sfi11virus* genus, mainly with the *Streptococcus* phage ALQ13.2.

Phage-host interactions were analyzed, considering the spacer acquisition in the CRISPR arrays. This finding demonstrated that twenty-seven spacers associated with the TP1-M17PTZA496, were predicted in three species of the genus *Streptococcus*, indicating mainly *S. thermophilus* (24 spacers) and, to a lesser extent, *S. salivarius* (2 spacers) and *S. macedonicus* (1 spacer) as the main potential hosts (Table S4).

### 3.2. Prophage Induction Evaluation

**Figure 4:**
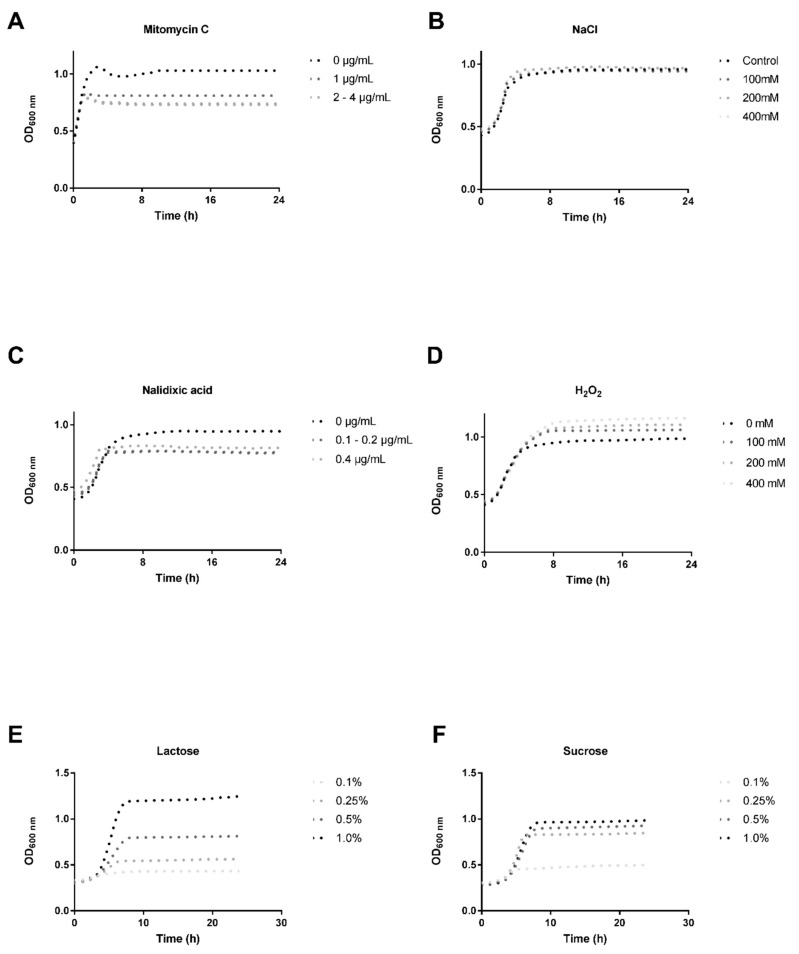
Phage-induction evaluation performed using four different chemicals: (**A**) Mitomycin C (1–4 µg/mL); (**B**) NaCl (100, 200, and 400 mM); (**C**) Nalidixic acid (0.1, 0.2, and 0.4 µg/mL); (**D**) H_2_O_2_ (100, 200, and 400 mM); (**E**) Lactose (0.1, 0.25, 0.5, and 1.0% *w*/*v*); (**F**) Sucrose (0.1, 0.25, 0.5, and 1.0% *w*/*v*). The same legend was used for different concentrations of the MmC (2–4 µg/mL) and the Nalidixic acid (0.1–0.2 µg/mL), since these growth curves overlapped each other.

The ability of the *S. thermophilus* M17PTZA496 to release inducible prophages was evaluated by treatment with four different phage-inducing agents (MmC, nalidixic acid, H_2_O_2_ and NaCl) added at the beginning of the log-phase (corresponding to OD_600_ ~ 0.3). It is worth mentioning that low concentrations of MmC (0.1, 02 and 0.5 µg/mL) were evaluated but no positive results were obtained [60]. Moreover, we used low (0.1, 0.25, and 0.5%) and high (1.0% *w*/*v*) concentrations of lactose and sucrose, in order to evaluate the effect of energy starvation on the bacteriophage induction. Comparison between the growth curves (24 h, 44 °C) of the treated and the untreated cultures, revealed that *S. thermophilus* M17PTZA496 possesses cryptic prophages. The presence of the prophages was clearly evidenced by the fact that the OD_600_ value in the treated samples, did not present significant decreases, which is typically represented by a growth inhibition, phage production, and host death (Figure 4). Based on these observations, we proceeded with further investigations, using only Mitomycin C.

**Figure 5:**
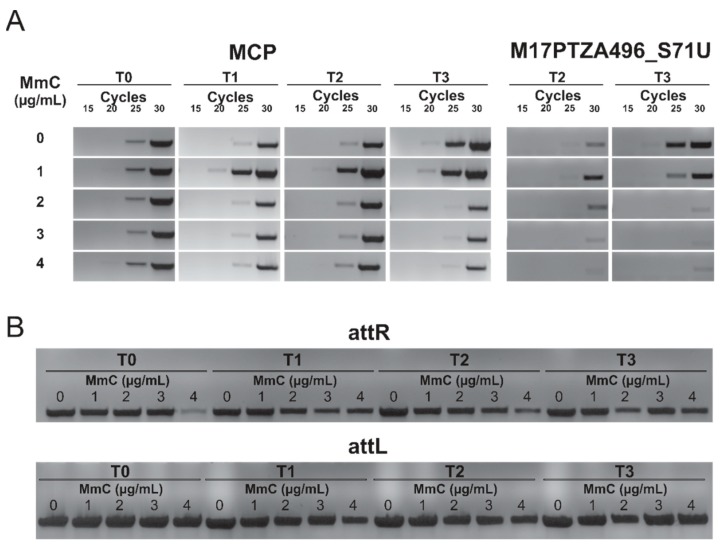
(**A**) Semi-quantitative PCR analysis showing the MCP levels at 30, 60, and 90 min, after addition of different amounts of MmC. M17PTZA496_S71U was used as the bacterial chromosome control region. (**B**) attL and attR sites amplification to check the absence of phage excision. T0, T1, T2, and T3 are the sampling times, namely 0, 30, 60, and 90 min, respectively under diverse MmC concentrations (1–4 µg/mL).

Additionally, in order to determine the copy number of the viral DNA in the lysogenic *S. thermophilus* M17PTZA496, semi-quantitative PCR analyses were performed, using the total genomic DNA as a template, and two different primer couples. Specific primers targeting the MCP gene were used to determine the prophage TP1-M17PTZA496 copy number, while primers targeting a genomic region of the bacterium (Scaffold 71; 297,265–304,822 bp) were used to determine the number of copies of the bacterial chromosome. Analyses were performed on bacteria cultures grown at different MmC concentrations and at multiple time points, after treatment (Figure 5A). Results showed that the MCP copy-number increased after 30 (T1) and 60 min (T2), following the MmC addition (1 µg/mL), as revealed by the presence of a detectable signal, after twenty amplification cycles. In fact, Arioli et al. (2018) [37] described the heterogeneity of *S. thermophilus* DSM20617^T^ determined by the phage excision events, even in the non-cured cells. On the other hand, when higher amounts of MmC were used (2, 3 and 4 µg/mL), a reduction in the MCP content was observed and a detectable signal was identified at the 25th cycle. As described by Oliveira et al. (2017) [41], high concentrations of MmC might have a toxic effect on the *Lactococcus lactis* culture harboring prophage and we observed the same phenomenon in the *S. thermophilus* M17PTZA496. In all the conditions tested, the complete sequences of the *att*L and the *att*R sites were identified (Figure 5B), confirming that prophage TP1-M17PTZA496 is not able to be completely excised from the host chromosome.

Finally, TEM analysis of the samples obtained from the treated and the untreated cultures was performed, considering four time points after exposure to MmC. Results were in agreement with the semi-quantitative PCR and revealed that no complete or defective phage particles were present in the lysogenic *S. thermophilus* M17PTZA496 strain.

### 3.3. S. thermophilus M17PTZA496 Phage-Susceptibility Assay

**Figure 6:**
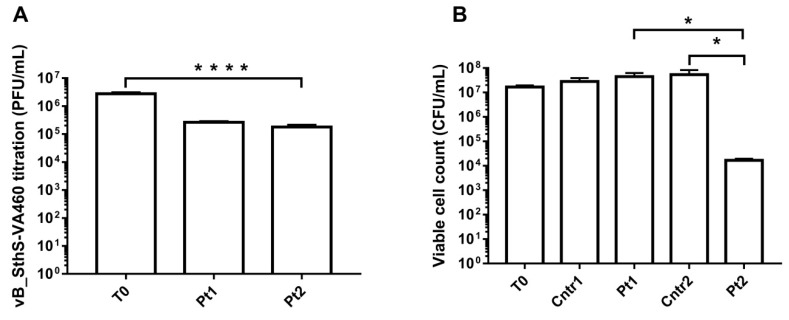
(**A**) Phage titration of the *S. thermophilus* cultures. Samples were taken at three different time points, namely at 0 (T0), 30 (Pt1), and 60 min (Pt2). (**A**) After addition of the VA460 plus MmC. (**B**) *S. thermophilus* M17PTZA496 viable cell counts, after PBS (Cntr1), VA460 (Pt1), Mitomycin C (Cntr2 and Pt2), and VA460 plus MmC (Pt2) addition. Asterisks indicate different levels of statistical significance (*: *p* ≤ 0.05; ****: *p* < 0.0001).

Six different *cos*-type bacteriophages, previously characterized in terms of their variable region (VR2), were used to perform phage-susceptibility screenings. *S. thermophilus* M17PTZA496 was only found to be susceptible but not permissive, to phage vB_SthS-VA460 (VA460), thus, suggesting the presence of bacterial mechanisms blocking it from starting the lytic cycle. After viral titration was performed on VA460, a 1-log reduction of the viral content was observed (90.4% of phages bound, Figure 6A). Measurement of the phage VA460 attachment rate was performed using the *S. thermophilus* M17PTZA496 cells as target, and it was calculated to be 7.79 × 10^−9^ mL/min.

To evaluate the effectiveness of *S. thermophilus* M17PTZA496 phage defense mechanisms, the expression level of *cas7*, *ltp*, and the ORF11 (Siphovirus Gp157 located in a viral *cro-ori* region), was investigated [61,62,63]. Additionally, to determine the capability of the prophage TP1-M17PTZA496 to package its genomic DNA, the expression level of the terminase large subunit gene was analyzed. RT-PCR analysis showed that MmC provided at a concentration of 1 µg/mL, led to a small but significant reduction in the expression level of both *cas7* and terminase large subunits (0.4 and 0.15-fold, respectively, Table 2). A 0.4-fold reduction was also observed for the *ltp*, Siphovirus Gp157, and high subunit expression levels, when only phage VA460 was added. Conversely, when MmC and VA460 were provided together, the *ltp* expression level increased 2.2-fold. A very similar result was obtained for the terminase large subunit gene (1.8 fold).

In accordance with the gene expression results, no reduction of the *S. thermophilus* M17PTZA496 viable cells was observed when the MmC or VA460 were independently added to the bacterial culture. Instead, a significant 3-log reduction (Figure 6B) was observed when *S. thermophilus* M17PTZA496 was exposed to a combination of the VA460 and the MmC.

## 4. Discussion

According to Hols et al. (2005) [64], HGT among bacteria contributes to the development of important industrially-relevant phenotypic characteristics. However, phage-mediated HGT is favored by the virus capability to infect different hosts [65,66], a quite uncommon feature for *S. thermophilus* bacteriophages, which are normally characterized by a narrow host spectrum [45,47]. An in silico host range prediction revealed that prophage TP1-M17PTZA496 has the potential capability to infect multiple *Streptococcus* species, including *S. thermophilus*, *S. macedonicus*, and *S. salivarius*. This interesting characteristic suggests that TP1-M17PTZA496 could have a potential role in the HGT.

Lysogeny is an infrequent process in *S. thermophilus* [67,68]. In the present study, the analysis of the prophage TP1-M17PTZA496 lysogeny module identified a putative *cI*-like repressor, while a gene coding for a *cro*-like repressor was absent. This is relevant to increase our knowledge of phage DNA replication properties and gene expression of the *S. thermophilus* phages, for which little information is currently available [21]. Cro and CI proteins mutually repress each other and are involved in the lytic/lysogenic switch [69,70]. As discussed below, TP1-M17PTZA496 is considered a non-inducible prophage and this behavior can be associated with the lack of a *cro*-like repressor [71]. Moreover, the terminase large subunit was flanked by genes involved in the lysis/morphogenesis and a group I intron was also identified in the CDS. The homing of group I introns are horizontally transferred, via mixed infections, as described for *pac*-type *Streptococcus* phage 2972, and removed through phage mRNA splicing [72]. Similar to the TP1-M17PTZA496, the phage 2972 possessed two group I intron sequences located in the genes coding for the terminase large subunit and for the endolysin. An alignment performed between the prophage M17PTZA496 and *Streptococcus* phage 2972 sequences, revealed that the same intron was present in the terminase large subunit of both viruses. This result corroborated our hypothesis that the prophage TP1-M17PTZA496 originated via mixed infection between the *cos*- and the *pac*-*S. thermophilus* phages. Despite the process that led to the classification of the TP1-M17PTZA496, as a mix between the *cos*- and the *pac*- *S. thermophilus* bacteriophages, phylogeny assigned it to the *S. thermophilus* group 3MSP-*pac*. This group includes other *pac*-type *Streptococcus* phages, such as O1205, 858, TP-J34, and Sfi11 [15,73,74,75,76].

Bioinformatics analysis revealed that the TP1-M17PTZA496 has interesting features associated with phage immunity, along with the absence of *cro*, in its lysogeny module. To verify this finding, the ability of the prophage to excise was checked, both using four different chemical compounds, and also by testing lactose- and sucrose-limiting conditions. The growth profiles of the treated cultures indicated that the TP1-M17PTZA496 can be considered non-inducible. Additional PCR-based and TEM analyses were in agreement in asserting that the TP1-M17PTZA496 can be considered a cryptic prophage with a very low excision rate, unable to form the phage particles. This result is consistent with previous findings indicating that different prophages in the *E. coli* K-12 can have very low excision rates, with values that may reach less than 1 prophage per 100,000 cells, or adopt the pseudo-lysogeny life cycle [37,73,77,78]. To further support results related to the excision ability, gene expression analyses were used. Since the small and large terminase subunits are components of the packaging machinery [79], their expression is representative of the tailed phages ability to pack their DNA. The large terminase relative expression analysis confirmed that TP1-M17PTZA496 has different activity levels, which slightly decreased after the MmC and VA460 treatments, but markedly increased, after the combined application of both stimuli. Interestingly, after the combined application, a marked reduction of the *S. thermophilus* M17PTZA496 viable cells was observed, although no cellular lysis was detected. This observation might be due to SOS system response activation and the phage-attack response, which justifies the absence of viable cells after plating.

Six *cos*-type *S. thermophilus* bacteriophages were screened for their capability to infect the *S. thermophilus* M17PTZA496. After viral titration, it was demonstrated that VA460 was able to adsorb *S. thermophilus* M17PTZA496, without provoking host lysis (rate of attachment 7.79 × 10^−9^ mL/min). Our result was in agreement with those of Bull et al. (2014) [80], who reported similar constant adsorption rate values (from 10^−8^ to 10^−9^ mL/min), as a result of the collision between phage and bacteria that culminated in viral infection, a relevant step to evaluate the bacterium self-defense mechanisms against phages. For this reason, VA460 was chosen for the phage immunity evaluation of *S. thermophilus* M17PTZA496. In particular, sie and host protection were tested by means of the Ltp type protein and the Siphovirus Gp157 expression.

A significant increase in the *ltp* expression (2.2 fold) was observed when the *S. thermophilus* M17PTZA496 was treated with a combination of VA460 and MmC. However, when only VA460 was added, a 0.4-fold decrease in expression (*p* < 0.05) was noticed. It was previously reported that *ltp* is transcribed only in the prophage state [15]; accordingly, we suggest that TP1-M17PTZA496 is a non-inducible prophage that actively expresses *ltp*. However, its low expression level could be attributed to the prophage transient excision activity level and, consequently, to the *ltp* expression reduction. It is worth mentioning that the relative expression level of each gene was normalized, using the *gapdh* gene of the *Mus musculus* as an external reference control. This normalization was needed considering that the addition of 1 µg/mL of MmC impaired the stable expression of the housekeeping genes *gapdh*, *recA*, and the *rpoD* of the *S. thermophilus* M17PTZA496 [60]. As described elsewhere [81,82], the use of an external reference gene is a valuable solution when the expression levels of the internal reference genes are not stable under determined conditions.

With regard to the short viral noncoding region, its function has been predicted to be similar to a 302-bp noncoding viral element (system PER—phage-encoded resistance) found in the *S. thermophilus* bacteriophage ΦSfi21 genome [83]. This region acts as an independent replication origin activated by viral infection and its action is copy-number dependent. In the present study, a 0.4-fold reduction in the number of copies of the Siphovirus Gp157 was observed in the presence of the phage VA460. This finding can be explained by the activity of this element, which is able to bind to the infected viral proteins and to decrease their free copy-number. As reported by Foley et al. (1998) [17], this region protected the *S. thermophilus* Sf1 from infection by seventeen out of the twenty-five evaluated phages. Noncoding *oris* regions were also previously reported in other bacteriophages, such as lactococcal phage P335 [84], *S. thermophilus* bacteriophages DT1, Sfi19, Sfi21, O1205, 7201, and κ3 [21].

Finally, the unexpected presence of two prophages in the *S. thermophilus* M17PTZA496 genome was previously hypothesized to be related to the low number of CRISPR-Cas modules found in this strain [28]. Relative expression of the *cas7*, a CRISPR protein involved in spacer acquisition [61], was examined with the aim of evaluating the influence of the bacterial adaptive immune system, under phage and antibiotic treatments. It was observed that only MmC influenced the *cas7* expression leading to a 0.4-fold reduction. The generally low activity of the CRISPR-Cas defense mechanism highlights the importance of the previously described mechanism in host superinfection exclusion. Moreover, using the *S. thermophilus* and the virulent phage 2972 as models, Vale et al. (2015) [85] suggested that the maintenance of the CRISPR-Cas defense system requires a great load of energy for the cell, that can result in decreased bacterial fitness. Since the MmC determines DNA damage and induces the SOS response, a process that requires a conspicuous amount of energy [86], it could be hypothesized that the MmC suppresses the bacterial adaptive immune system, in order to save energy.

## 5. Conclusions

Although the CRISPR-Cas system plays a relevant role in the phage-host defense, the presence of cryptic prophages can enhance bacterial defenses. Here, we reported the occurrence of Ltp and a noncoding viral DNA element in the non-inducible prophage TP1-M17PTZA496. Thanks to these additional immune mechanisms, *S. thermophilus* M17PTZA496 can be proposed as an alternative model to study bacterial resistance to phages. In conclusion, considering the enormous importance of this bacterial species for the industrial production of fermented foods, *S. thermophilus* M17PTZA496 could be considered as a promising alternative to the phage-sensitive starter cultures for the manufacturing of dairy products.

## Figures and Tables

**Table 1:** Primers used for semiquantitative PCR and RT-qPCR assays.

Primer	Sequence (5’ → 3’)	Reference	Genome position (bp)
PhageCntr_FW	CCAGCTCGCAAACAACTTGG	This study	644,332–645,130
PhageCntr_REV	CAGCGTTAACTGTGTTGTCAG		
*attL*_FW	CACGCTGCTAACTCAATCCT	This study	620,880–621,469
*attL*_REV	GCTCTTTGGATATCCACACC		
*attR*_FW	CTACGTAGTCAGAGGTCCG	This study	664,158–664,626
*attR*_REV	GATTAAAGGCCTATTCTAAGCC		
M17ptza496_S71U_FW	GCAACCATTACACACATAAGGT	This study	297,265–304,822
M17ptza496_S71U_REV	CACAGCGACATCTATCATTGG		
*cas7*_FW_1	AGGAGCCTACCATACTTGATG	This study	697,396–698,448
*cas7*_REV_1	GTAAGCGTGGGCAAGTGTTC		
*ltp*_FW_4	ACTAGCAAGACGTCAGAGGC	This study	697,265–697,399
*ltp*_REV_4	CTGCTTAGCTTTCTCACCG		
NC_FW_5	CAACTTACAGACCAGACAAGG	This study	626,422–626,895
NC_REV_5	CCTCAATATGCTTACCGGAC		
Terminase large subunit_FW_2	CATGGTGCTAAACGTGCTGG	This study	639,113–639,784
Terminase large subunit_REV_2	GCAGGTACATCGTCAACGTC		
*gapdh*_FW	CACCATCTTCCAGGAGCGAG	Conte et al. (2015) [43]	(not determined)
*gapdh*_REV	CACCATCTTCCAGGAGCGAG		

**Table 2:** Relative expression of the cas7, ltp, Siphovirus Gp157, and terminase large subunit. Only statistically significant values (*p* < 0.05) were considered. (Cntr1—PBS; Pt1—VA460; Cntr2—MmC; Pt2—VA460 plus MmC).

	*cas7*		*ltp*		Siphovirus Gp157		Terminase Large Subunit	
Conditions	*p*-value	X-fold	*p*-value	X-fold	*p*-value	X-fold	*p*-value	X-fold
Cntr1 vs Pt1	0.093	0.50	0.012	0.40	0.016	0.40	0.012	0.40
Pt2 vs Cntr2	0.154	1.80	0.022	2.20	0.756	1.10	0.026	1.75
Cntr1 vs Cntr2	0.025	0.40	0.320	0.70	0.250	0.80	0.000	0.15

## Supplementary Materials

The following are available online at http://www.mdpi.com/1999-4915/11/1/7/s1, Figure S1: RT-qPCR experimental design, Figure S2: RNA integrity was evaluated on denaturing formaldehyde agarose gel (0.5% *w*/*v*), Figure S3, PHASTER analysis indicating the two prophages (positions 617,680–669,542 and 890,763–919,156) integrated in the *S. thermophilus* M17PTZA496 chromosome, named (A) TP1-M17PTZA496 and (B) TP2-M17PTZA496, Table S1: Genbank accession number of the bacteriophages sequences (or genome sequences?) used to construct the phylogenomic tree, Table S2: TP2-M17PTZA496 annotation considering three different databases. Asterisks indicate lack of information according to each database, Table S3: 61 ORFs were predicted among *attL* and *attR* attachment sites, Table S4: 27 different spacers were predicted for the prophage M17PTZA496 in 3 diverse species of the genus *Streptococcus* (24 S. thermophilus, 2 S. salivarius and 1 S. macedonicus) using the CRISPRdb database.
