# Supplementary material for: A Cryptic Non-Inducible Prophage Confers Phage-Immunity on the Streptococcus thermophilus M17PTZA496
Source: Viruses. 2018 Dec 22;11(1):7. doi: 10.3390/v11010007 (PMC6356513; doi:10.3390/v11010007)

Supplementary Table S1 – Genbank accession number of the bacteriophages sequences (or genome sequences?) used to construct the phylogenomic tree. A total of 83 *S. thermophilus* phages and, 5 *Lactococcus lactis* phages were used to compose the tree.

**Genbank accession number**	**Bacteriophage**
NC 002747	*Lactococcus* phage TP901-1
NC 002703.1	*Lactococcus* phage Tuc2009
DQ838728.1	*Lactococcus* phage P335
DQ394806.1	*Lactococcus* phage ul36.k1
NC 004066.1	*Lactococcus* phage ul36
KX879642.1	*Streptococcus* phage CHPC926
KX879641.1	*Streptococcus* phage CHPC577
NC 013598.1	*Streptococcus* phage ALQ13.2
NC 007019.1	*Streptococcus* phage 2972
NC 010353.1	*Streptococcus* phage 858
NC 022776.1	*Streptococcus* phage TP-778L
NC 002214.1	*Streptococcus* phage Sfi11
NC 020197.1	*Streptococcus* phage TP-J34
NC 004303.1	*Streptococcus* phage O1205
NC 000871.1	*Streptococcus* phage Sfi19
NC 000872.1	*Streptococcus* phage Sfi21
NC 002072.2	*Streptococcus* phage DT1
NC 013645.1	*Streptococcus* phage Abc2
NC 002185.1	*Streptococcus* phage 7201
NC 012753.1	*Streptococcus* phage 5093
KX879643.1	*Streptococcus* phage CHPC1151
CM002372.1	*S. thermophilus* M17PTZA496
NC_023503.1	*Streptococcus* phage 20617
NC_031094.1	*Streptococcus* virus 9872
NC_031069.1	*Streptococcus* virus 9871
MH000602	*Streptococcus* phage D5842
MH000603.1	*Streptococcus* phage D1024
MF161328	*Streptococcus* phage D4276
MH000604	*Streptococcus* phage D1811
KY705258.1	*Streptococcus* phage P4761
KY705268.1	*Streptococcus* phage P7571
KY705269.1	*Streptococcus* phage P7572
KY705277.1	*Streptococcus* phage P7951
KY705278.1	*Streptococcus* phage P7952
KY705279.1	*Streptococcus* phage P7953
KY705280.1	*Streptococcus* phage P7954
KY705281.1	*Streptococcus* phage P7955
KY705285.1	*Streptococcus* phage P9852
KY705286.1	*Streptococcus* phage P9853
KT717085.1	*Streptococcus* phage 128
MF580763.1	*Streptococcus* phage 16B8
MF580764.1	*Streptococcus* phage 31B4
KT717084.1	*Streptococcus* phage 53

KT717083.1	*Streptococcus* phage 73
MF580759.1	*Streptococcus* phage 7A5
MF580760.1	*Streptococcus* phage 7T
MF580761.1	*Streptococcus* phage 9A
MF580762.1	*Streptococcus* phage 9B4
MF580765.1	*Streptococcus* phage A0
MF580766.1	*Streptococcus* phage B0
MF580767.1	*Streptococcus* phage B5
MF580768.1	*Streptococcus* phage C0
MH000603.1	*Streptococcus* phage D1024
MH000604.1	*Streptococcus* phage D1811
MF161328.1	*Streptococcus* phage D4276
MH000602.1	*Streptococcus* phage D5842
MF580769.1	*Streptococcus* phage L5A1
MF580770.1	*Streptococcus* phage M19
MF580771.1	*Streptococcus* phage MM25
KY705251.1	*Streptococcus* phage P0091
KY705256.1	*Streptococcus* phage P3681
KY705257.1	*Streptococcus* phage P3684
KY705259.1	*Streptococcus* phage P5641
KY705260.1	*Streptococcus* phage P5651
KY705261.1	*Streptococcus* phage P5652
KY705262.1	*Streptococcus* phage P7132
KY705263.1	*Streptococcus* phage P7133
KY705264.1	*Streptococcus* phage P7134
KY705265.1	*Streptococcus* phage P7151
KY705266.1	*Streptococcus* phage P7152
KY705267.1	*Streptococcus* phage P7154
KY705270.1	*Streptococcus* phage P7573
KY705271.1	*Streptococcus* phage P7574
KY705272.1	*Streptococcus* phage P7601
KY705273.1	*Streptococcus* phage P7602
KY705274.1	*Streptococcus* phage P7631
KY705275.1	*Streptococcus* phage P7632
KY705276.1	*Streptococcus* phage P7633
KY705282.1	*Streptococcus* phage P8921
KY705283.1	*Streptococcus* phage P8922
KY705284.1	*Streptococcus* phage P9851
KY705287.1	*Streptococcus* phage P9854
KY705288.1	*Streptococcus* phage P9901
KY705289.1	*Streptococcus* phage P9902
KY705290.1	*Streptococcus* phage P9903
MF580772.1	*Streptococcus* phage R1
MF580773.1	*Streptococcus* phage STP1
MF580774.1	*Streptococcus* phage STP2
MF580775.1	*Streptococcus* phage V2
MG708274.1	*Streptococcus* phage vB_SthS_VA214
MG708275.1	*Streptococcus* phage vB_SthS_VA460
MG708273.1	*Streptococcus* phage vB_SthS_VA698
NC_018285.1	*Streptococcus* phage YMC-2011

Supplementary Table S2. TP2-M17PTZA496 annotation considering three different databases. Asterisks indicate lack of information according to each database

	Phaster		Uniprot			Pfam	
Position	Best hit	E-Value	Best hit	E-Value	Identity	Best hit	Accession [illegible]
890763.. 890775	*attL*	N/A	*	*	*	*	*
891288.. 891321	*attL*	N/A	*	*	*	*	*
896847.. 897140)	Integrase core domain protein	1.71e-18	Transposase	2.9e-61	99.0%	DDE_Tnp_IS240	PF1361[illegible]
897152.. 897442	Transposase	3.89e-09	Integrase core domain protein	1.1e-55	91.8%	HTH_32	PF1356[illegible]
897805.. 898287	Sensory protein	N/A	Carotenoid biosynthetic protein CrtK	1.3e-110	100.0%	TspO_MBR	PF0307[illegible]
898485.. 898862	Hypothetical protein	N/A	NADH:ubiquinone oxidoreductase 27 kD subunit	8.9e-78	95.9%	Lipoprotein_22	PF1729[illegible]
899394.. 899603	Hypothetical protein	N/A	Eps4Q	2.3e-9	96.2%	*	*
899501.. 899731	Putative transposase A	8.01e-09	Transposase	2.4e-55	100.0%	HTH_Tnp_1	PF01527
900252.. 900325	tRNA	N/A	*	*	*	*	*

900428..900628	Hypothetical protein	1.03e-07	Bacteriocin	7.2e-50	98.5%	*	*
900759..900959	Csp	2.09e-17	Cold-shock protein A	2.3e-50	100.0%	CSD	PF00313
901239..901439	Csp	8.42e-37	Cold shock protein B	3.3e-49	100.0%	CSD	PF00313
902102..902135	*attR*	N/A	*	*	*	*	*
902208..904349	Copper-translocating P-type ATPase	N/A	Copper-translocating P-type ATPase	0.0	96.7%	E1-E2_ATPase	PF00122
904858..905184	Restriction endonuclease	N/A	Type I restriction endonuclease	1.2e-74	100.0%	*	*
905181..905814	Putative restriction-modification protein	5.24e-08	Type I restriction-modification system methyltransferase subunit	1.0e-123	99.4%	N6_Mtase	PF02384
905731..905982	ISL3 family transposase	N/A	Transposase	1.2e-40	81.0%	*	*
906294..906305	*attL*	N/A	*	*	*	*	*
906321..907208	Sugar transporter	N/A	Sugar transporter	0.0	100.0%	Sugar_transport	PF06800

907282.. 907465	Hypothetical protein	N/A	DD-carboxypeptidase PBP3 Serine peptidase	9.3e-14	81.6%	*	*
908277.. 909109	Nuclease	N/A	Nuclease	0.0	100.0%	*	*
909115.. 909708	Integrase	3.10e-21	Integrase	9.5e-140	100.0%	Phage_integrase	PF00589
909785.. 910816	Hypothetical protein	N/A	Uncharacterized protein	0.0	98.0%	*	*
910773.. 910967	Hypothetical protein	N/A	Uncharacterized protein	8.6e-45	96.9%	*	*
911116.. 911484	Hypothetical protein	N/A	Dipeptidase	1,00E-84	100.0%	Peptidase_C69	PF03577
911540.. 912349	PrsW family intramembrane metalloprotease	N/A	Uncharacterized protein	2.8e-15	50%	*	*
912507.. 914051	Zinc ABC transporter substrate-binding protein AdcA	N/A	Zinc ABC transporter substrate-binding protein	0.0	100.0%	ZinT	PF09223
914135.. 914146	*attR*	N/A	*	*	*	*	*

914268.. 914666	Hypothetical protein	N/A	Uncharacterized protein	2.4e-90	100.0%	*	*
914827.. 915127	Hypothetical protein	N/A	Uncharacterized protein	7.2e-34	95.2%	*	*
915182.. 916189	Putative sulfate exporter family transporter	N/A	Putative sulfate exporter family transporter	0.0	98.5%	Cons_hypoth6 98	PF03601
916362.. 917075	Lysin	1.08e-14	N-acetylmuramidase	5.6e-170	100.0%	Glucosaminida se	PF01832
917087.. 917317	Transposase	N/A	Transposase	2.3e-59	100.0%	*	*
917478.. 917958	Transposase B	2.75e-28	IS3-Spn1, transposase	1.1e-94	100.0%	rve	PF00665
919156.. 919168	*attR*	N/A	*	*	*	*	*

Supplementary Table S3 – 61 ORFs were predicted among *attL* and *attR* attachment sites. In the total, 17 ORFs remained as coding sequences for hypothetical proteins. TP1-M17PTZA496 annotation was performed using three different approaches. Asterisks indicate lack of information according to each database.

ORF	MyRast prediction	Functional categorization	Start	Stop	Strand	PFAM prediction	E-Value	PFAM accession	Uniprot prediction	UNIPROT accession
1	Phage integrase	Lysogeny module	1266	103	-	Phage integrase family	6.8e-23	PF00589.21	*	*
2	Hypothetical protein	Immunity	1843	1415	-	Host cell surface-exposed lipoprotein	6.6e-15	PF07553.10	*	*
3	Phage protein	Conjugation/transposon exchange	2276	1890	-	ImmA	3.6e-09	PF06114.12	*	*
4	Putative cI-like repressor-phage associated	Repressor/Lysogeny module	2604	2266	-	*	*	*	*	*
5	Hypothetical protein	Replication	2803	3015	+	*	*	*	DNA-binding protein (*S. thermophilus* M17PTZA496)	A0A0E2Q201
6	Hypothetical protein	*	3045	3989	+	*	*	*	*	*
7	Phage Antirepressor protein	Repressor/Lysogeny module	3982	4527	+	ORF6C domain	5.4e-07	PF10552.8	**	**
8	Phage excisionase	Recombination	4533	4772	+	*	*	*	*	*
9	Phage protein	*	4916	5185	+	*	*	*	*	*
10	Hypothetical protein	*	5204	5362	+	*	*	*	*	*
11	Phage protein	Immunity	5543	6016	+	Siphovirus Gp157	6.4e-37	PF05565.10	*	*
12	Phage DNA-binding protein	Replication	6013	6714	+	AAA proteins	2.2e-30	PF13479.5	*	*
13	DNA helicase, phage-associated	Replication	6689	8020	+	Type III restriction enzyme, res subunit	8.8e-11	PF04851.14	*	*
14	Single stranded DNA-binding protein, phage-associated	Replication	8027	8482	+	Protein of unknown function (DUF669)	1.1e-22	PF05037.12	*	*
15	Phage replication protein	Replication	8485	9291	+	Bifunctional DNA primase/polymerase, N-terminal	1.5e-25	PF09250.10	*	*
16	DNA Primase/helicase, phage-associated	Replication	9278	10792	+	Poxvirus D5 protein-like	2.6e-18	PF03288.15	*	*
17	Phage protein	*	11047	11478	+	*	*	*	*	*
18	Conserved hypothetical protein - phage associated	*	11460	11780	+	VRR-NUC domain	4.8e-11	PF08774.10	*	*
19	Phage protein	*	11764	12006	+	*	*	*	*	*
20	Hypothetical protein	*	11991	12170	+	*	*	*	*	*

21	Hypothetical protein	*	12167	12388	+	*	*	*	*	*
22	Phage protein	*	12378	12536	+	*	*	*	*	*
23	Adenine-specific methyltransferase (EC 2.1.1.72)	*	12529	12813	+	*	*	*	*	*
24	Adenine-specific methyltransferase (EC 2.1.1.72)	*	12843	13280	+	DNA methyltransferase	2.8e-19	PF01555.17	*	*
25	Hypothetical protein	*	13336	13827	+	Protein of unknown function (DUF1642)	9.2e-29	PF07852.10	*	*
26	Hypothetical protein	*	14016	14297	+	*	*	*	*	*
27	Hypothetical protein	*	14319	14900	+	Protein of unknown function (DUF1642)	1.3e-16	PF07852.10	*	*
28	Phage DNA-binding protein	*	14901	15413	+	Helix-turn-helix	1.0e-11	PF01381.21	*	*
29	Phage protein	*	15388	15690	+	Protein of unknown function (DUF1372)	4.8e-39	PF07116.10	*	*
30	Hypothetical protein	*	15687	16100	+	YopX protein	2.3e-22	PF09643.9	*	*
31	Phage protein	*	16102	16809	+	Protein of unknown function (DUF1340)	2.7e-139	PF07067.10	*	*
32	Phage protein	Host lysis	17197	17613	+	*	*	*	Autolysin (*S. thermophilus* M17PTZA496)	A0A0E2Q2R7
33	Phage terminase, small subunit	Packaging	17795	18247	+	Terminase small subunit	3.2e-18	PF03592.15	*	*
34	Phage terminase, large subunit	Packaging	18273	18905	+	*	*	*	*	*
35	Phage terminase, large subunit	Packaging	19193	19780	+	Terminase RNaseH-like domain	8.5e-08	PF17289.1	*	*
36	Phage portal protein	Head morphogenesis	19789	21294	+	Phage portal protein, SPP1 Gp6-like	1.3e-104	PF05133.13	*	*
37	Phi Mu50B-like protein	Head morphogenesis	21291	22184	+	Phage Mu protein F like protein	6.6e-24	PF04233.13	*	*
38	Phage capsid and scaffold	Head morphogenesis	22375	22956	+	Domain of unknown function (DUF4355)	1.1e-16	PF14265.5	*	*
39	Phage major capsid protein	Head morphogenesis	22976	23335	+	*	*	*	*	*
40	Phage major capsid protein	Head morphogenesis	23354	24400	+	Phage major capsid protein E	6.7e-40	PF03864.14	*	*
41	Phage protein	*	24402	24563	+	*	*	*	*	*
42	Phage protein	Structural protein	24575	24916	+	Phage gp6-like head-tail connector protein	7.9e-06	PF05135.12	*	*
43	Phage protein	*	24913	25227	+	*	*	*	*	*
44	Phage protein	*	25229	25573	+	*	*	*	Histone H1(*S. thermophilus* M17PTZA496)	A0A0E2Q247
45	Phage capsid and scaffold	*	25570	25956	+	Protein of unknown function (DUF3168)	4.5e-05	PF11367.7	*	*
46	Phage tail protein	Tail morphogenesis	25969	26478	+	Phage tail tube protein	5.5e-27	PF06199.10	*	*
47	Phage protein	Tail morphogenesis	26551	26904	+	Phage tail assembly chaperone protein, TAC	5.9e-30	PF12363.7	*	*
48	Phage protein	*	26994	27272	+	*	*	*	*	*
49	Phage tail length tape-measure protein	Tail morphogenesis	27262	31899	+	*	*	*	*	*
50	Phage tail protein	Tail morphogenesis	31899	33434	+	Phage tail protein	8.1e-38	PF05709.10	*	*

51	Phage tail protein	Tail morphogenesis	33437	36913	+	Prophage endopeptidase tail	2.3e-13	PF06605.10	*	*
52	Phage capsid and scaffold	Structural protein	36914	38962	+	Siphovirus protein of unknown function (DUF859)	9.5e-299	PF05895.11	*	*
53	Phage protein	*	38989	39399	+	Protein of unknown function (DUF1366)	8.8e-51	PF07104.10	*	*
54	Phage protein	*	39419	39565	+	*	*	*	*	*
55	Hypothetical protein	*	39578	39901	+	*	*	*	*	*
56	Phage holin	Host lysis	39909	40151	+	Bacteriophage holin	6.8e-17	PF04531.12	*	*
57	Phage lysin, N-acetylmuramoyl-L-alanine amidase (EC 3.5.1.28)	Host lysis	40153	40998	+	N-acetylmuramoyl-L-alanine amidase	5.3e-15	PF05382.12	*	*
58	Hypothetical protein	*	41657	42001	+	*	*	*	*	*
59	Phage protein	*	42023	42574	+	*	*	*	Elongation factor G (*S. thermophilus* M17PTZA496)	A0A0E2Q264
60	Hypothetical protein	*	42662	42979	+	*	*	*	*	*
61	Phage protein	*	43013	43435	+	*	*	*	*	*

Supplementary Table S4 – 27 different spacers were predicted for the prophage M17PTZA496 in 3 diverse species of the genus *Streptococcus* (24 *S. thermophilus*, 2 *S. salivarius* and 1 *S. macedonicus*) using the CRISPRdb database. Only spacers with E-Value less than 0.001 are shown.

<table>
<tr><th>Sequence ID</th><th>Spacer sequence</th><th>Identity</th><th>E-Value</th></tr>
<tr><td>S. thermophilus JIM 8232</td><td>gttctatccattcagcgataacttagaagaattaaaa
|||||||||||||||||||||||||||||||||||||
gttctatccattcagcgataacttagaagaattaaaa</td><td>1.0</td><td>2.00E-10</td></tr>
<tr><td>S. thermophilus JIM 8232</td><td>ttaactggatttggtcaaacaagaaaacagaaacagt
||||||||||||||||||||||| |||||||||||||
ttaactggatttggtcaaacaagcaaacagaaacagt</td><td>0.9</td><td>3.00E-08</td></tr>
<tr><td>S. thermophilus ASCC 1275
S. thermophilus MN-BM-A02
S. thermophilus MN-BM-A0
S. thermophilus KLDS SM
S. thermophilus ND07</td><td>cgcaacccctccttagacatgggaacagtacta
|||||||||||||||||||||||||||||||||
cgcaacccctccttagacatgggaacagtacta</td><td>1.0</td><td>3.00E-08</td></tr>
<tr><td>S. thermophilus ASCC 1275
S. thermophilus MN-BM-A02
S. thermophilus KLDS SM
S. thermophilus ND07</td><td>atattgttaatgataactacaaatttaaacgag
|||||||||||||||||||||||||||||||||
atattgttaatgataactacaaatttaaacgag</td><td>1.0</td><td>3.00E-08</td></tr>
<tr><td>S. thermophilus ND03</td><td>cggacagcgataaatacactctatacagaga
|||||||||||||||||||||||||||||||
cggacagcgataaatacactctatacagaga</td><td>1.0</td><td>3.00E-07</td></tr>
<tr><td>S. thermophilus CNRZ1066
S. thermophilus CS8</td><td>taatcgtaaagaggttttggaatacgttct
||||||||||||||||||||||||||||||
taatcgtaaagaggttttggaatacgttct</td><td>1.0</td><td>1.00E-06</td></tr>
<tr><td>S. thermophilus LMD-9
S. thermophilus ASCC 1275
S. thermophilus MN-BM-A02
S. thermophilus SMQ-301
S. thermophilus KLDS SM</td><td>catcataggcggaactggtaggatgtacac
||||||||||||||||||||||||||||||
catcataggcggaactggtaggatgtacac</td><td>1.0</td><td>1.00E-06</td></tr>
<tr><td>S. thermophilus JIM 8232</td><td>aagaagcagcaagatgcatcaaacaccgca
||||||||||||||||||||||||||||||
aagaagcagcaagatgcatcaaacaccgca</td><td>1.0</td><td>1.00E-06</td></tr>
<tr><td>S. thermophilus JIM 8232
S. thermophilus ASCC 1275
S. thermophilus MN-BM-A02
S. thermophilus KLDS SM
S. thermophilus ND07</td><td>ttcttccgttcttcaaacgttaaattccaa
||||||||||||||||||||||||||||||
ttcttccgttcttcaaacgttaaattccaa</td><td>1.0</td><td>1.00E-06</td></tr>
<tr><td>S. thermophilus MN-ZLW-002
S. thermophilus MN-BM-A01</td><td>caacccagacatgaatgtcattagatatgt
||||||||||||||||||||||||||||||
caacccagacatgaatgtcattagatatgt</td><td>1.0</td><td>1.00E-06</td></tr>
<tr><td>S. thermophilus ASCC 1275
S. thermophilus MN-BM-A02
S. thermophilus KLDS SM
S. thermophilus ND07</td><td>gagcgagctcgaaataatcttaattacaag
||||||||||||||||||||||||||||||
gagcgagctcgaaataatcttaattacaag</td><td>1.0</td><td>1.00E-06</td></tr>
<tr><td>S. thermophilus ASCC 1275
S. thermophilus MN-BM-A02
S. thermophilus KLDS SM
S. thermophilus ND07</td><td>cgagtaagtattaatcaggattgggacgcc
||||||||||||||||||||||||||||||
cgagtaagtattaatcaggattgggacgcc</td><td>1.0</td><td>1.00E-06</td></tr>
</table>

<table>
<tr><td>S. thermophilus ASCC 1275
S. thermophilus MN-BM-A02
S. thermophilus KLDS SM
S. thermophilus ND07</td><td>aatgacgaggagctattggcacaacttaca
||||||||||||||||||||||||||||||
aatgacgaggagctattggcacaacttaca</td><td>1.0</td><td>1.00E-06</td></tr>
<tr><td>S. thermophilus ND07</td><td>catcataggcggaactggtaggatgtacac
||||||||||||||||||||||||||||||
catcataggcggaactggtaggatgtacac</td><td>1.0</td><td>1.00E-06</td></tr>
<tr><td>S. salivarius CCHSS3</td><td>tgaatctgtccagtcttcagtgtctagcaagg
||||||||||||||||||| ||||||||||||
tgaatctgtccagtcttcattgtctagcaagg</td><td>0.9</td><td>1.00E-05</td></tr>
<tr><td>S. thermophilus JIM 8232</td><td>taaagaacataaacatataatgaatactttagaact
|||||||||||||||||| |||||| ||||||||||
taaagaacataaacatatgatgaatgctttagaact</td><td>0.9</td><td>1.00E-05</td></tr>
<tr><td>S. thermophilus JIM 8232</td><td>aaacattgatgagtattattgataaacttca
|||||||||||||||||||||| ||||||||
aaacattgatgagtattattgaaaaacttca</td><td>0.9</td><td>5.00E-05</td></tr>
<tr><td>S. thermophilus KLDS 3.1003</td><td>tgaaagcactatggaacgatacattagtagt
|||||||||||||||| ||||||||||||||
tgaaagcactatggaatgatacattagtagt</td><td>0.9</td><td>5.00E-05</td></tr>
<tr><td>S. thermophilus LMD-9
S. thermophilus SMQ-301</td><td>aacagttactattaatcatgattccaacgg
|||||||||||||||||| |||||||||||
aacagttactattaatcacgattccaacgg</td><td>0.9</td><td>2.00E-04</td></tr>
<tr><td>S. thermophilus JIM 8232</td><td>tatatataagtttccgaaagtaaacgaacc
|||||||||||||||||||||| |||||||
tatatataagtttccgaaagtagacgaacc</td><td>0.9</td><td>2.00E-04</td></tr>
<tr><td>S. thermophilus JIM 8232</td><td>ggacgcatattgaacaacataacccattta
|||||||||||||||| |||||||||||||
ggacgcatattgaacagcataacccattta</td><td>0.9</td><td>2.00E-04</td></tr>
<tr><td>S. salivarius 57.I</td><td>ttcgaaatatttatattttccatttggtaa
||||||||||||||||||||| ||||||||
ttcgaaatatttatattttccgtttggtaa</td><td>0.9</td><td>2.00E-04</td></tr>
<tr><td>S. thermophilus MN-ZLW-002
S. thermophilus ASCC 1275
S. thermophilus MN-BM-A02
S. thermophilus MN-BM-A01
S. thermophilus KLDS SM
S. thermophilus ND07</td><td>cgatggaaatgatggattgccaggtaagga
||||||||||||||| ||||||||||||||
cgatggaaatgatggcttgccaggtaagga</td><td>0.9</td><td>2.00E-04</td></tr>
<tr><td>S. macedonicus ACA-DC 198</td><td>ggctttccgtcaaacattacgactttttc
||||| |||||||||||||||||||||||
ggcttaccgtcaaacattacgactttttc</td><td>0.9</td><td>6.00E-04</td></tr>
<tr><td>S. thermophilus JIM 8232</td><td>aaaag-tgttgacaaactatcatatatg
||||| ||||||||||||||||||||||
aaaaggtgttgacaaactatcatatatg</td><td>0.9</td><td>6.00E-04</td></tr>
<tr><td>S. thermophilus ASCC 1275
S. thermophilus MN-BM-A02
S. thermophilus KLDS SM
S. thermophilus ND07</td><td>aatcaatacgacaagag-taaacatggtctt
||||||||||||||||| |||| ||||||||
aatcaatacgacaagagttaaa-atggtctt</td><td>0.9</td><td>6.00E-04</td></tr>
<tr><td>S. thermophilus KLDS 3.1003</td><td>aatgtcatggggttctgatatgtctagct
||||||||||||| |||||||||||||||
aatgtcatggggtgctgatatgtctagct</td><td>0.9</td><td>6.00E-04</td></tr>
</table>

Supplementary Figure S1. RT-qPCR experimental design. Cntr1 – PBS buffer; Pt1 – phageVA460; Cntr2 – MmC (1 µg/mL); Pt2 – VA460 plus MmC (1 µg/mL). T0, T1 and T2 are, respectively, sampling times after 0, 30 and 60 min.

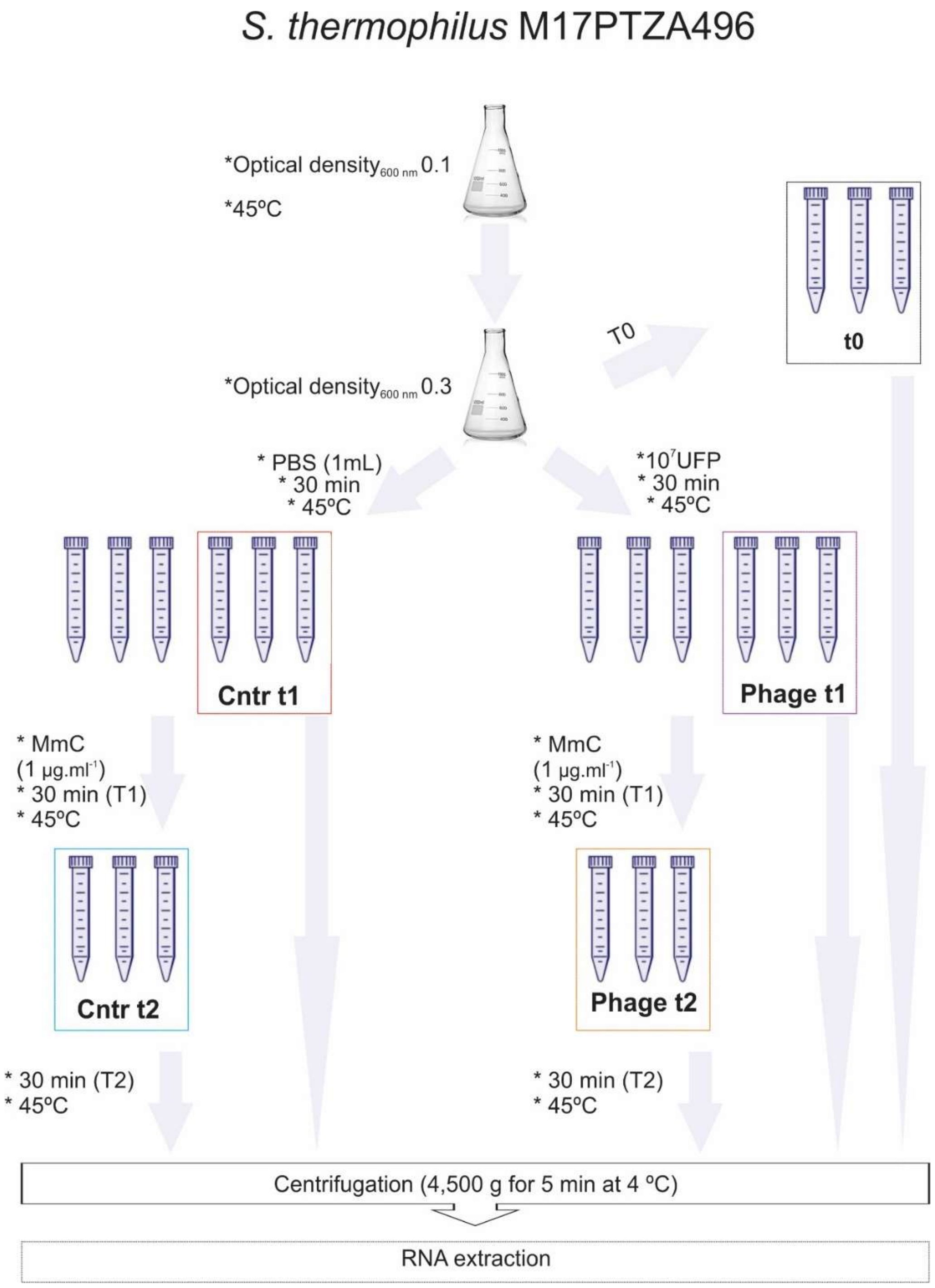

Supplementary Figure S2 –RNA integrity was evaluated on denaturing formaldehyde agarose gel (0.5% w/v). We used as positive control (C+) RNA extracted from *Saccharomyces cerevisiae*. Samples were taken after three different times: 0 (T0), 30 (Pt1) and 60 min (Pt2). Cntr1 and Cntr2 correspond, respectively, sampling after PBS buffer and Mitomycin C addition.

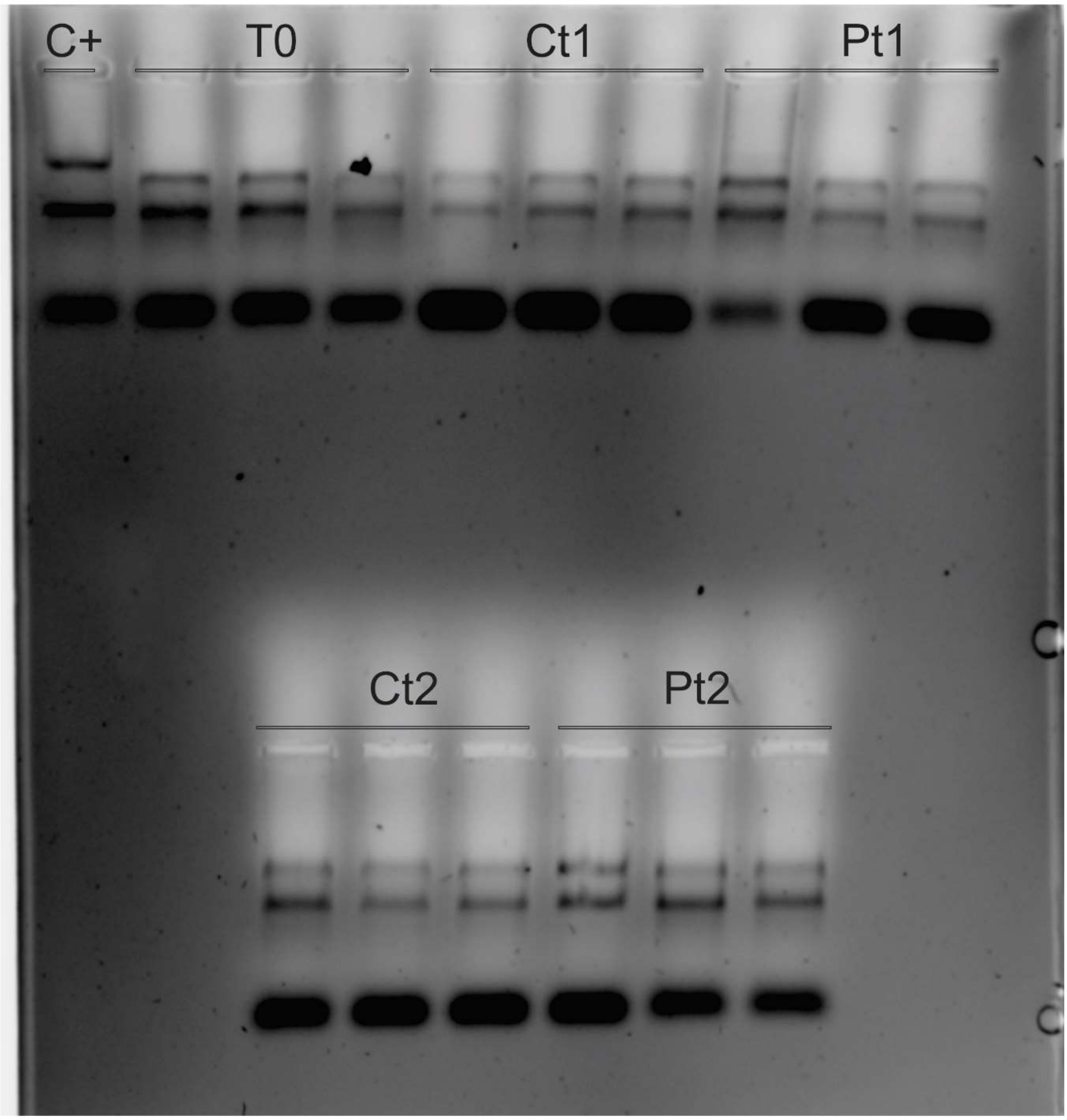

Supplementary Figure S3 – PHASTER analysis indicating the two prophages (positions 617,680 - 669,542 and 890,763 - 919,156) integrated in the *S. thermophilus* M17PTZA496 chromosome, named (A) TP1-M17PTZA496 and (B) TP2-M17PTZA496. PLP – Phage-like protein; Att – Attachment site; Hyp – Hypothetical protein; Ter – Terminase; Por – Portal protein; Coa – Head protein; Sha – Tail protein; Int – Integrase; Oth – Non phage-like protein; Tra – Transposase; RNA - tRNA. Left-right arrow indicates virus DNA “plus strand”, whereas right-left corresponds to viral DNA “minus strand”.

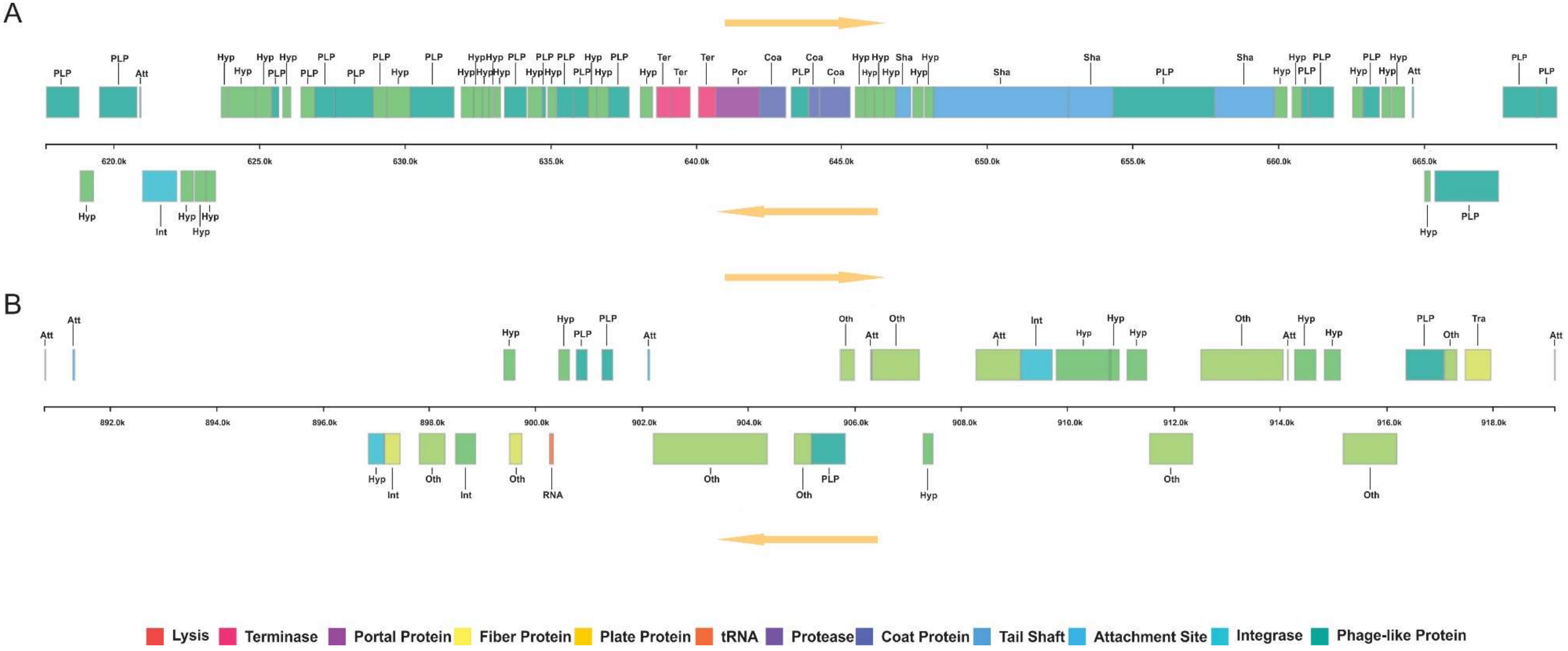

Supplement: Supplementary file 1 [file viruses-11-00007-s001.pdf]
